# Prevalence of Depressive Symptoms Amongst Pregnant Women Attending Antenatal Clinics at Quaternary Hospital in Johannesburg, South Africa: A Cross-Sectional Study

**DOI:** 10.3390/ijerph22091446

**Published:** 2025-09-18

**Authors:** Ugasvaree Subramaney, Lawrence Chauke

**Affiliations:** 1 Department of Psychiatry, School of Clinical Medicine, Faculty of Health Sciences, University of the Witwatersrand, Johannesburg 2000, South Africa; ugasvaree.subramaney@wits.ac.za; 2 Department of Obstetrics and Gynecology, School of Clinical Medicine, Faculty of Health Sciences, University of the Witwatersrand, Johannesburg 2000, South Africa; 3 Charlotte Maxeke Johannesburg Academic Hospital, Johannesburg 2193, South Africa

**Keywords:** antenatal depression, high risk pregnancy, EPDS, prevalence

## Abstract

Antenatal depression significantly contributes to maternal and neonatal morbidity worldwide; however, the rate of screening, particularly in low- and middle-income countries (LMICs), remains very low. This cross-sectional survey study was aimed at determining the prevalence of depressive symptoms among women aged 18 to 34 years attending antenatal clinics at a quaternary hospital in Johannesburg, South Africa, utilizing a Biographical Questionnaire and the Edinburgh Postnatal Depression Scale (EPDS). The study is based on a total of 151 questionnaires. The mean age of the study population was 27.6 years (range: 18–34). Majority of participants identified themselves as Black (138, 91.4%), had previously been pregnant (111, 73.5%), were in the third trimester of pregnancy (89, 58.9%), were unemployed or seeking employment (108, 71.5%), and had no pre-existing medical (107, 70.9%) or mental illnesses (143, 94.7%). The prevalence of antenatal depressive symptoms among the study population was 43.7% (66/151), and 18 (27.3%) of the women who screened positive had suicidal ideation. The prevalence of antenatal depressive symptoms in this study exceeds that reported in other regions, underscoring the urgent need for universal screening throughout pregnancy and provision of perinatal mental healthcare services for pregnant and postpartum women.

## 1. Introduction

Antenatal depression, defined as the occurrence of depressive symptoms during pregnancy, is one of the most common psychiatric disorders of pregnancy, affecting a substantial number of pregnant women [1]. This is different from perinatal depression, which is usually used to refer to depression that begins from the moment of conception, continuing for up to 12 months after childbirth [2]. Antenatal depression, which is more prevalent than postnatal depression, is increasingly recognized as a significant public health problem globally [1,2,3]. However, further studies are needed specifically in low- and middle-income countries (LMICs), where the prevalence may be higher due to the greater presence of associated risk factors, particularly socio-economic challenges [1,4].

In South Africa, the prevalence has been reported to range between 21% and 48.9% with higher rates reported amongst rural populations [4,5,6]. The variations in prevalence reported in these studies might be due to the different screening tools employed, the availability of resources, and social stressors present in different communities [1,2,3,4,5,6]. The diagnosis should always be considered in pregnant women who present with persistent low mood, anxiety, fatigue, and emotional withdrawal [1].

Factors contributing to antenatal depression include low socioeconomic status, unemployment, low levels of education, unplanned and unwanted pregnancies, a history of substance abuse, lack of social support or a supportive partner, personal history of mental illness, childhood trauma, current or historical intimate partner violence, previous pregnancy complications—including pregnancy loss—and recent significant adverse social events [1,2,3,4,5,6,7,8].

Antenatal depression has both short- and long-term adverse effects on the affected women and their offspring. In the short term, the impacts include poor self-care and antenatal care attendance, as well as an increased risk of pregnancy complications such as preterm labor [9,10]. If left untreated, affected women face a higher risk of postnatal depression, suicide, including psychiatric illnesses in the long term [11,12]. The impact of untreated antenatal depression on affected women cannot be ignored. Untreated antenatal depression increases the risk of maternal suicide. Between 2005 and 2022, suicide accounted for 39% of the maternal deaths in the USA [13]. According to the 2020–2022 Mothers and Babies: Reducing Risk through Audits and Confidential Enquiries across the UK (MBRRACE) report, suicide accounted for 39% of maternal deaths between 6 weeks and 12 months after birth, and Black women were four affected when compared to their white counterparts [14]. Similarly, suicide is among the top three leading causes of maternal deaths in the USA [13].

Similarly, suicide is emerging as a significant contributing factor to maternal mortality in South Africa [15,16]. Before the 2014–2016 Saving Mothers report, maternal deaths due to suicide were classified under medical and surgical conditions, obscuring the contribution of suicide to maternal mortality. However, the National Confidential Committee on Maternal Deaths (NCCEMD) established a separate category for suicide after it became apparent that it is an important contributing factor to maternal deaths in South Africa. According to the South African Saving Mother maternal deaths reports, 74 women died by suicide during the 2017–2019 triennial period, and the majority had no personal history of psychiatric illness [16]. The same report suggests that this figure is probably an underestimation due to the lack of a comprehensive system to capture maternal deaths occurring outside healthcare facilities. Furthermore, some suicide-related maternal deaths may have been misclassified under other categories, including medical and surgical, including maternal deaths resulting from the acute collapse of unknown cause.

Antenatal depression has significant implications for offspring development, both during and beyond the pregnancy period. During pregnancy, the condition is associated with complications such as intrauterine growth restriction (IUGR), low birth weight (LBW), and neonatal intensive care unit (NICU) admission. Additionally, children exposed to maternal depression during pregnancy are at an increased risk of physical, motor, and cognitive developmental delays, emotional dysregulation, and long-term behavioral problems [17,18,19,20,21,22]. It has been suggested that maternal antenatal depression may disrupt fetal neurodevelopment by affecting the maternal hypothalamic–pituitary–adrenal (HPA) axis and causing epigenetic modifications in the fetal brain, predisposing children to problematic and antisocial behavior later in life [23,24]. A meta-analysis of perinatal mental health and child development identified associations between impaired social-emotional functioning, reduced verbal and performance IQ, and increased externalizing behaviors throughout childhood and adolescence [25]. These findings emphasize the intergenerational impact of untreated maternal depression, reinforcing the urgency of integrating mental health services into routine antenatal care to safeguard the well-being of these mothers’ offspring.

Treatment involves a combination of psychological, pharmacological, and lifestyle interventions. The most utilized non-pharmacological treatments, cognitive behavioral therapy (CBT) and interpersonal therapy (IPT), have been shown to be effective in reducing depressive symptoms [25]. However, pharmacological treatment, such as selective serotonin reuptake inhibitors (SSRIs), may be necessary for moderate to severe cases, including those that do not respond to non-pharmacological interventions [26]. When implemented early, the interventions can help mitigate both the short- and long-term negative impacts of antenatal depression on both mothers and their offspring.

There exist different recommendations regarding screening for antenatal depression during pregnancy. The American College of Obstetricians and Gynecologists (ACOG) advocates for routine screening as part of antenatal care at least once, using validated tools such as the Edinburgh Postnatal Depression Scale (EPDS) or Patient Health Questionnaire 9 (PHQ-9) [27]. Similarly, the National Institute for Health and Care Excellence (NICE) in the UK encourages healthcare workers to routinely inquire about mental health during antenatal visits, followed by structured screening if there are concerns [28]. In contrast, the Canadian Task Force on Preventive Health Care recommends targeted screening, particularly for individuals with known risk factors [29].

Several validated instruments are used to screen for antenatal depression, each with distinct advantages depending on the clinical context and available resources. The EPDS is the most widely recommended tool for perinatal populations, as it excludes somatic symptoms that may complicate the diagnosis of antenatal depression during pregnancy [30,31,32]. Its brevity (10 items) and strong psychometric properties across diverse settings (sensitivity ~0.81, specificity ~0.87) further support its use [30]. In contrast, general depression scales such as PHQ-9, Beck Depression Inventory-II (BDI-II), and Centre for Epidemiologic Studies Depression Scale (CES-D) include physical symptoms common in pregnancy, which may lead to an overestimation of depressive severity [31,32]. A validation study conducted in South Africa, comparing the EPDS, PHQ-9, K10, K6, and Whooley questions, found EPDS to be the most accurate and contextually appropriate tool for low-resource settings [33]. Furthermore, the EPDS has been validated for use in LMICs settings like South Africa, and its endorsement by professional bodies such as the ACOG and the Perinatal Mental Health Partnership (PMHP) provides a strong case for its integration into routine antenatal care [26,27].

Despite emerging evidence that suicide is one of the causes of maternal mortality in South Africa [15,16], maternal mental health has yet to be integrated into antenatal care. Furthermore, the limited local studies conducted on this topic have primarily focused on low-risk pregnant women in primary healthcare settings. This has resulted in a significant gap in research regarding the prevalence of antenatal depression among high-risk women, particularly those receiving care in tertiary and quaternary hospitals, such as the study site, where prevalence may be higher in the presence of comorbidities. Currently, there are no perinatal mental healthcare services provided at the study site. Consequently, this study aimed to determine the prevalence of depressive symptoms among women attending antenatal clinics at a quaternary hospital in Johannesburg, South Africa, and to utilize this information to inform clinical practice and government maternal healthcare policy.

## 2. Materials and Methods

### 2.1. Study Design

This is cross-sectional survey of pregnant women attending an antenatal clinic, utilizing self-administered piloted Biographical Questionnaire (including, amongst others, age, employment, population group, medical history, habits, socioeconomic status, number of children) and the Edinburgh Postnatal Depression Scale (EPDS) Questionnaire. The EPDS is the most commonly used depression screening tool in perinatal care. It consists of 10 items, scored between 0 and 4 to indicate the severity and frequency of depressive symptoms in the last week. A cut-off value of 10 or higher indicates possible depression, and 13 or higher probable depression. The survey was performed over four months in 2019, before the COVID-19 pandemic.

### 2.2. Study Site

The study was conducted at a quaternary academic hospital in Johannesburg, Gauteng Province, South Africa. The Department of Obstetrics and Gynecology at this facility serves as a referral center for high-risk pregnancies, supporting four district hospitals, six regional hospitals, and ten midwifery obstetric units across three healthcare districts, namely, City of Ekurhuleni, City of Johannesburg, and the West Rand District. It also serves patients from the neighboring provinces of Mpumalanga and Northwest, as well as self-referrals from African countries including Zimbabwe, Mozambique, Malawi, Congo, Lesotho, and Sudan. The maternity unit at the study site conducts approximately 11,000 deliveries annually.

### 2.3. Study Population and Sample

The study population comprises all pregnant women attending antenatal clinics at the study site. The study sample consisted of a convenience sample of pregnant women who provided informed consent and completed the study questionnaire.

### 2.4. Data Collection

Data on maternal characteristics and pregnancy-related information were collected using a specially designed Biographical Questionnaire and the EPDS for depression screening. The self-administered questionnaires were piloted with four participants who were not involved in the study, and no modifications were required. Each morning, women attending antenatal clinics were briefed by research assistants while they waited for their appointments and were invited to participate. Those who consented received written informed consent forms and questionnaires to complete, which they returned to the research assistants who were always on-site to answer any questions. The women participating in the study were not compensated for their participation. All de-identified data were stored on an Excel spreadsheet on a password-protected computer, and access was limited to the research team after the data had been collected by the research assistants.

### 2.5. Data Analysis

Data analysis was conducted using Microsoft Power BI^®^, which facilitated enhanced visualization of the data.

### 2.6. Ethics Considerations

Permission to conduct the study was obtained from the Chief Executive Officer (CEO) at the study site and ethics clearance from the Human Research Ethics Committee of the University of Witwatersrand (Ethics reference: M181078). All study participants provided informed written consent prior to their participation.

## 3. Results

### 3.1. Prevalence and Profile of Study Participants

Demographic and pregnancy characteristics of the study sample are summarized in Table 1. A total of 151 (42.9%) out of the 352 questionnaires issued were returned and were included in the analysis. The mean age of the study population was 27.6 years (range: 18–34). The majority of the participants (138, 91.4%) identified as Black African, had a history of previous pregnancies (111, 73.5%), were in the third trimester of their pregnancies (89, 58.9%), were unemployed or seeking employment (108, 71.5%), and had no pre-existing medical (107, 70.9%) or mental health (143, 94.7%) conditions. Eighty-eight participants (58.3%) had no family history of medical or mental illness, while 139 (92.1%) were satisfied with their pregnancies. Among the 111 participants (73.5%) who had experienced prior pregnancies, 36 (32.4%) had a history of miscarriage, the majority (21, 58.3%) of which occurred during the third trimester. The average income within the study population was R9534.00 (range R400 to R50,000). Fifteen (10.0%) and eight (5.3%) participants consumed alcohol and smoked cigarettes, respectively. Using a cut-off of 13 on EPDS, 66 out of the 151 participants (43.7%) screened positive for antenatal depressive symptoms on the EPDS Questionnaire. The cut-off score of 13 was chosen because of its higher specificity [13]. The majority of the women who screened positive for depressive symptoms were in their first (27, 40.9%) and third (21, 31.8%) trimesters, indicating a bimodal distribution of depressive symptoms. The remaining cases accounted for 18 (27.2%). Unmarried status and dissatisfaction with pregnancy were common among those who screened positive for antenatal depressive symptoms.

### 3.2. Risk of Suicidality and Thoughts of Self-Harm

The risk of suicidality and thought of self-harm are summarized in Table 2. Participants who screened positive for depressive symptoms were stratified according to their level of suicidal risk: low (score of 0), at risk (scores of 1, 2, and 3, with 3 indicating the highest risk), using Question 10 on the EPDS, which assesses the risk for suicidality and self-harm. Among the 66 women, 18 (27.3%) screened positive for suicidal risk, with six (33.3%) classified as level 1, three (16.7%) as level 2, and nine (50.0%) as level 3. Of the six classified under level 1 suicidal risk, four (66.7%) were in their third trimester, and three (50.0%) had never been married and expressed dissatisfaction with their pregnancies. Among those categorized as level 2, two (66.7%) were in their second trimester and one (33.3%) in their third trimester; none were ever married or were unhappy with their pregnancies. For the nine women classified under level 3, five (55.6%) had never been married, two (22.2%) were in their first trimester, and three (33.3%) indicated dissatisfaction with their pregnancies.

## 4. Discussion

Antenatal depression is a significant global public health concern; however, routine screening and treatment remain inadequate, particularly in LMIC. Van Heyningen et al. [33] reported a 75–80% treatment gap for mental health disorders among pregnant women. This condition poses serious public health risks, including increased maternal mortality. The aim of this study was to assess the prevalence of depressive symptoms among women with high-risk pregnancies attending antenatal clinics at a quaternary hospital, utilizing the EPDS Questionnaire alongside demographic and pregnancy-related inquiries. The sensitivity, specificity, and cost effectiveness of EPDS as a screening tool for antenatal and postnatal depression were confirmed in a systematic review by Chorwe-Sungani and Chipps, making it attractive for limited resource settings [34]. This screening tool is easy and quick to use and has been validated in South Africa using the international agreed cut-off of 13 [5].

In this study, the prevalence of depressive symptoms was 43.7%. The majority of the women identified as Black South Africans had pre-existing comorbidities, were young, with a mean age of 27.6 years, and had no personal or family history of mental illness. Twenty-seven per cent of those who screened positive for depressive symptoms also screened positive for suicidal risk. Unmarried status and dissatisfaction with pregnancy were common among those who screened positive for antenatal depressive symptoms. The predominantly Black African cohort reflects the patient demographic typical of the study site. In South Africa, the Black population group is considered vulnerable due to historical factors, including a higher unemployment rate, which is a known risk factor for antenatal depression [35]. Similarly, the mean age of 27.3 aligns with the inclusion criteria of 18–34 years. Pregnant women under the age of 18 and those over the age of 35 were excluded from the study due to the reported higher incidence of antenatal depression, which is associated with increased morbidity and stress linked to unplanned pregnancies in these age groups [1,4,5,6,36].

The prevalence of 43.7% reported in this study is significantly higher than the 7–20% reported in high-income countries [2] and the 21.5% reported in Ethiopia [1], as well as the 39.4% observed in Zimbabwe, which primarily involved HIV-positive women [21]. However, the figure is lower than the 47% and 48.9% reported by Rochal et al. [5] and Vythilingum et al. [37], respectively. The variation in the prevalence of antenatal depressive symptoms across different studies likely reflects differences in the populations studied. Furthermore, the high prevalence in our study may be attributed to selection bias and the focus on high-risk women, 70.9% of whom had comorbidities. Rochal et al. concentrated on HIV-positive women, while Vythilingum employed an Edinburgh Postnatal Depression Scale (EPDS) cutoff score of 12, which diverges from the internationally recommended threshold. Unlike other studies that focused on either the first or third trimester of pregnancy and primary healthcare settings [1,2,5,21,37], our study included women across all three trimesters of pregnancy and was conducted in a quaternary setting, thereby contributing to the existing literature on the subject.

The study’s finding of a bimodal distribution (with the highest incidence of depressive symptoms during the first and second trimester) aligns with existing literature [38]. This finding further underscores the importance of screening for antenatal depression throughout pregnancy. Although the study did not specifically assess risk factors, some participants exhibited known risk factors for antenatal depression, including unemployment, a previous history of poor pregnancy outcomes, comorbidities, and a history of substance use. These factors are recognized as significant contributors to the risk of developing depression during pregnancy and the postpartum period [1,3,4,5,6,7,8]. Unmarried status and dissatisfaction with pregnancy were common among women who screened positive for antenatal depression, including an increased risk for suicidal ideation. Factors such as younger age, unemployment, immigrant status, a history of previous miscarriage, unplanned and unwanted pregnancies, and lack of partner support are acknowledged risk factors associated with increased maternal morbidity and mortality, including a heightened risk of suicidal ideation [8,33,35,36,37,38,39].

## 5. Limitations

This cross-sectional study was conducted over a limited timeframe using a convenience sample of participants, which restricts the generalizability of the findings. Furthermore, the study does not establish causality and does not investigate associated risk factors. Conducted within a hospital setting, the results may not be applicable to all levels of the healthcare system within Gauteng Province or South Africa as a whole. Additionally, as the questionnaire was self-administered, it may have been prone to both underestimation and overestimation of the prevalence of antenatal depression. We also recognize the potential for selection bias, given the response rate of 42.9%. Despite these limitations, the study emphasizes the importance of screening for antenatal depression during pregnancy and postpartum period. It also underscores the necessity for further research among high-risk pregnant women to gain a deeper understanding of the magnitude of the issue. A multicenter study could yield additional valuable insights.

## 6. Conclusions

The prevalence of antenatal depressive symptoms within the study population exceeds that reported in other studies from high-income and other low-income countries. This finding underscores the urgent need for universal screening of antenatal depression throughout pregnancy, along with the provision of perinatal mental healthcare services during pregnancy and the postpartum period. Furthermore, women who screen positive for antenatal depressive symptoms should undergo assessment for suicidal risk, followed by appropriate interventions.

## Figures and Tables

**Table 1:** Participant profile.

Variable (n = 151)	SD or Frequency
Mean age	27.6 (SD 4.5)
Population group	
Black African	138 (91.4%)
Colored	6 (3.9%)
White	3 (1.9%)
Indian/Asian	2 (1.3%)
Not stated	2 (1.3%)
Employment status	
Employed	43 (28.5%)
Unemployed	52 (34.4%)
Seeking employment	56 (37.1%)
Income per month (South African Rands)	
Minimum	400.00
Maximum	50.000
Average	9534.00
Preexisting medical condition	44 (29.1%)
No	107 (70.9%)
Yes	44 (29.1%)
Hypertension	5
HIV	4
Other (Diabetes, Asthma, Epilepsy, Collagen vascular diseases, etc.)	35 (79.4%)
Preexisting mental illness	
No	143 (94.7%)
Yes	8 (5.3%)
Depression	7 (87.5%)
Other	1 (12.5%)
Treatment	5 (62.5%)
Family history of medical/mental illness	
No	88 (58.3%)
Yes	63 (41.7%)
Trimester at study entry	
First	12 (7.9%)
Second	50 (33.1%)
Third	89 (58.9%)
Previous pregnancy	
No	11 (73.5%)
Yes	11 (73.5%)
Previous miscarriage	
No	36 (32.4%)
Yes	40 (26.5%)
Timing of previous miscarriage (n = 36)	
First trimester	3 (8.3%)
Second Trimester	12 (33.3%)
Third trimester	21 (58.3%)
Alcohol and smoking	
Alcohol	15 (10.0%)
Smoking	8 (5.3%)

**Table 2:** Risk of suicide and thoughts of self-harm.

Participants Who Tested Positive for Depressive Symptoms (n = 66)	Frequency
Suicidality risk	
No	48 (72.7%)
Yes	18 (27.3%)
Level	
Level 1	6 (33.3%)
Level 2	3 (16.7%)
Level 3	9 (50.0%)

## Data Availability

Data for the study are included in the manuscript. Any further information can be obtained from the authors.
